# The Effects of Physiological Biomechanical Loading on Intradiscal Pressure and Annulus Stress in Lumbar Spine: A Finite Element Analysis

**DOI:** 10.1155/2017/9618940

**Published:** 2017-08-27

**Authors:** Siti Nurfaezah Zahari, Mohd Juzaila Abd Latif, Nor Raihanah Abdull Rahim, Mohammed Rafiq Abdul Kadir, Tunku Kamarul

**Affiliations:** ^1^Faculty of Mechanical Engineering, Universiti Teknikal Malaysia Melaka, Hang Tuah Jaya, 76100 Durian Tunggal, Melaka, Malaysia; ^2^Center for Robotics and Industrial Automation (CeRIA), Universiti Teknikal Malaysia Melaka, Hang Tuah Jaya, 76100 Durian Tunggal, Melaka, Malaysia; ^3^Medical Devices and Technology Group, Faculty of Biosciences and Medical Engineering, Universiti Teknologi Malaysia, 81310 Skudai, Johor, Malaysia; ^4^Tissue Engineering Group (TEG), National Orthopaedic Centre of Excellence in Research and Learning (NOCERAL), Department of Orthopaedic Surgery, Faculty of Medicine, University of Malaya, Lembah Pantai, 50603 Kuala Lumpur, Malaysia

## Abstract

The present study was conducted to examine the effects of body weight on intradiscal pressure (IDP) and annulus stress of intervertebral discs at lumbar spine. Three-dimensional finite element model of osseoligamentous lumbar spine was developed subjected to follower load of 500 N, 800 N, and 1200 N which represent the loads for individuals who are normal and overweight with the pure moments at 7.5 Nm in flexion and extension motions. It was observed that the maximum IDP was 1.26 MPa at L1-L2 vertebral segment. However, the highest increment of IDP was found at L4-L5 segment where the IDP was increased to 30% in flexion and it was more severe at extension motion reaching to 80%. Furthermore, the maximum annulus stress also occurred at the L1-L2 segment with 3.9 MPa in extension motion. However, the highest increment was also found at L4-L5 where the annulus stress increased to 17% in extension motion. Based on these results, the increase of physiological loading could be an important factor to the increment of intradiscal pressure and annulus fibrosis stress at all intervertebral discs at the lumbar spine which may lead to early intervertebral disc damage.

## 1. Introduction

Obesity has been recognised as a factor that could lead to chronic low back pain (LBP). This problem is expected to further escalate in the near future with the current increasing numbers of overweight and obese population [1, 2]. It was demonstrated that the increase of body weight will increase the stress at the lumbar spine which leads to potential factor of intervertebral disc (IVD) degeneration [3–6]. Furthermore, excessive loading applied on the lumbar spine tends to fracture the vertebral body endplate before damaging the IVD [7].

In computational studies, follower load applied to the lumbar spine could increase intradiscal pressure (IDP), intersegmental rotation and facet joint force [8, 9]. The compressive load on the spine reduces the disc height due to the decrease of the volume of mass gelatinous in nucleus pulposus. As the fluid is being squeeze out from the disc, the tissue will reorganize which caused the viscoelastic annulus collagen fibers to creep [10, 11]. Consequently, this increases the hydrostatic pressure and the outer annulus starts to bulge. Although these phenomenons have been described in many computational and clinical studies, the fundamental understanding that underpins the biomechanics leading to disc damage has yet to be explored. Furthermore, the relationship between the increase in body weight to the stresses occurs at various vertebral segments of the lumbar spine when the body in different posture needs to be elucidated.

In the present study, the effects of physiological loading on the lumbar spine were studied at all vertebral segments to examine the IVD during flexion and extension motions using finite element method. The IDP at nucleus pulposus and the von Mises stress (VMS) at annulus fibrosis of the IVD were investigated.

## 2. Materials and Methods

### 2.1. Finite Element Modeling

**Figure 1:**
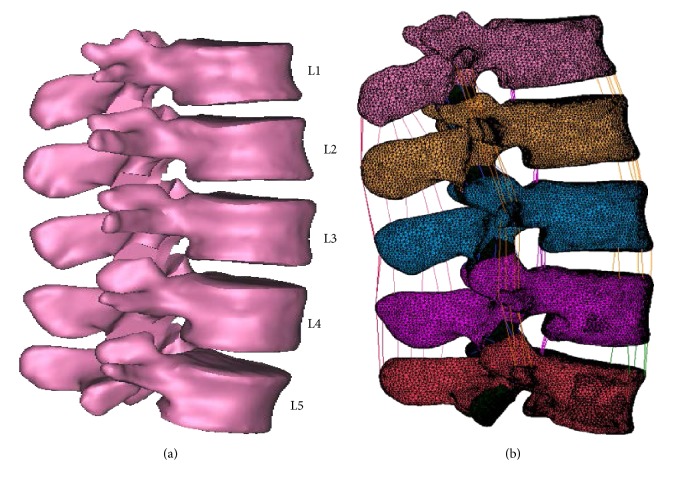
L1–L5 lumbar spine. (a) Three-dimensional model and (b) finite element model.

The geometrical data of lumbar vertebrae were obtained from computed tomography (CT) scan of a healthy 21-year-old male with 1.73 m height and 70 kg weight. The CT scan images of 3 mm slice thickness in two-dimensional (2D) Standard Tessellation Language (STL) format were segmented to develop a three-dimensional (3D) model of human lumbar spine using Mimics 14.0 (Materialise, Leuven, Belgium) and Magics (Materialise, Leuven, Belgium) softwares as shown in Figure 1(a). Marc Mentat 2011 (MSC, Software, Santa Ana, CA) finite element (FE) software was then used to generate the FE model using linear first-order tetrahedral elements as shown in Figure 1(b).

The vertebra was divided into hard cortical bone on the outside and less dense cancellous bone inside where linear isotropic material properties were imposed for both cortical and cancellous bones [12]. The thickness of cortical was set at 1 mm [13].

The 3D model of the IVD was created manually using SolidWorks (Dassault Systèmes SolidWorks Corporation) software where the volumetric ratio between the annulus and nucleus was set to 3 : 7 [14]. The top and the bottom surfaces of the disc were constructed such that the surfaces were in contact with the corresponding adjacent surfaces of the vertebral body using Mimics software. The IVD was composed of nucleus pulposus and annulus fibrosis which was modelled as hyperelastic using Mooney-Rivlin formulation [15, 16]. The annulus was constructed to be composite of a homogenous ground substance reinforced by collagen fibers. The fibers were represented by 3D truss element with nonlinear stress-strain curve material properties and the angle varied from ±24° to ±46° [12, 15].

The facet cartilage area was set as hyperelastic using the Mooney-Rivlin formulation with the thickness of 2 mm [12, 16]. The articulating facet surfaces were modelled as surface-to-surface contact with 0.5 mm initial gap where the normal contact stiffness was 200 N/mm and the friction coefficient is zero [15]. This will only allow the compressive force to be transmitted within the gap between the articulating facet surfaces [15, 17].

The ligaments were represented using truss elements.  Table 1 shows the geometrical parameter of the lumbar spine ligaments [15, 18]. The complete list of the material properties imposed in the FE model of the osseoligamentous lumbar spine is presented in Table 2.

Mesh convergence analysis was performed in order to obtain an optimum FE model of the lumbar spine. Four FE models of L4-L5 lumbar segment were developed using 1.5 mm, 2.0 mm, 2.5 mm, and 3 mm mesh sizes. The analysis was based on the IDP results of the IVD where the optimum mesh size started at 2 mm as the IDP reached a plateau value. The 2.0 mm was then applied in FE model of L1–L5 lumbar spine [12].

### 2.2. Finite Element Analysis

**Figure 2:**
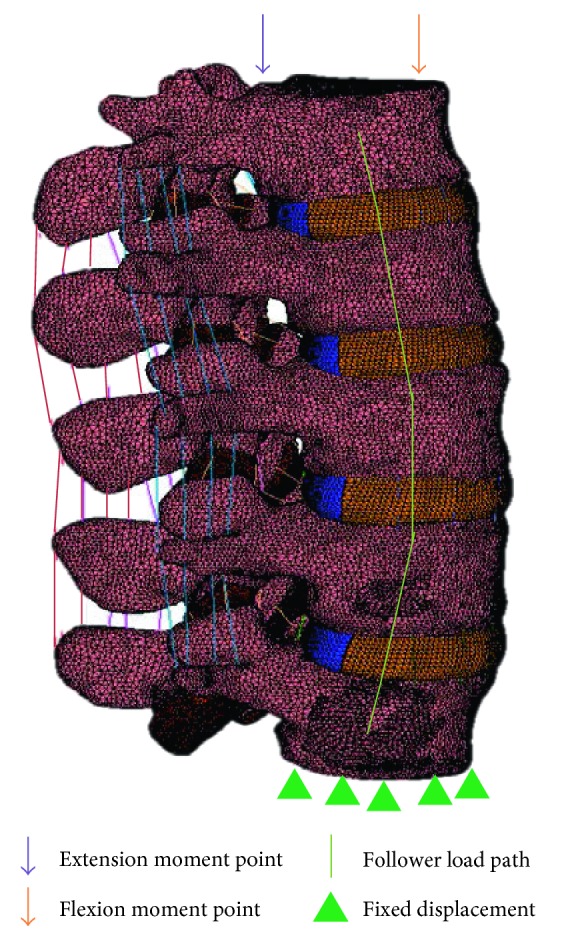
Loading and boundary condition of FE model of the lumbar spine.

The contact surfaces between the vertebral bodies and the IVD were set as perfectly connected to each other using segment to segment contact algorithm in Marc Mentat software. The FE model was subjected to follower load of 500 N, 800 N, and 1200 N which represent the typical human normal weight, overweight, and obesity based on 65% of upper body weight with an additional 200 N of local muscle force [19, 20]. Pure moment of 7.5 Nm was generated using force couple applied at flexion and extension moment points (Figure 2) to create either flexion or extension motions [21, 22]. The force couple consists of two equal and opposite forces as shown in Table 3 [23].

Eight spring elements were applied around the L1–L5 lateral vertebral body where the total load was divided equally to each of the spring element [24, 25]. This is to assure the uniformity of the applied follower load and to avoid any potential rotation of the intervertebral body. The inferior surface of the L5 vertebral body was completely fixed in all directions as shown in Figure 2.

## 3. Results

### 3.1. Verification of FE Model

**Figure 3:**
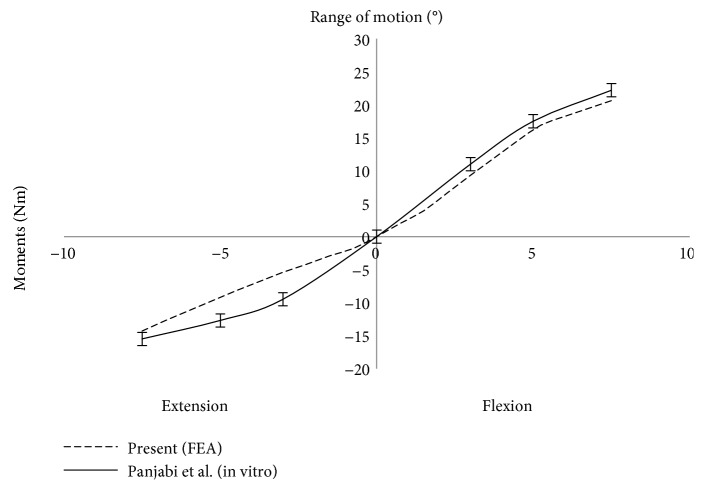
Comparison of ROM between present FE model and previous in vitro result under 7.5 Nm pure moments.

The FE model of osseoligamentous lumbar spine was verified by comparing the range of motion (ROM) with previous in vitro study for flexion and extension motions at pure moment of 7.5 Nm. The present results of the intersegmental rotations of the lumbar spine follow similar trend to the previous in vitro results as shown in Figure 3 [21]. The percentage difference of the ROM between present and previous in vitro study in flexion was 7.5% at 7.5 Nm. Although notable difference was found between 2 Nm and 5 Nm in extension motion of the lumbar spine, the percentage difference of the ROM was decreased to 8.1% when reaching 7.5 Nm moments.

**Figure 4:**
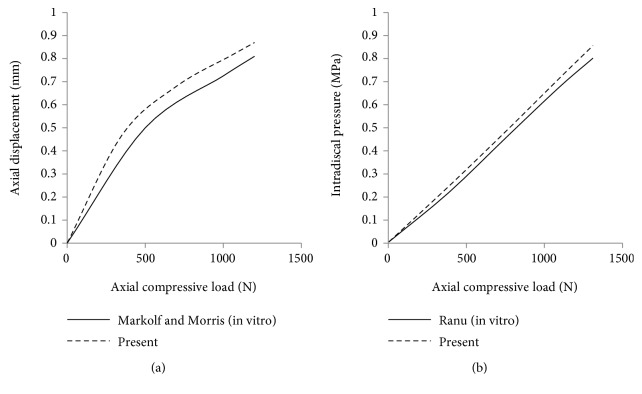
Comparison of present FEA and previous in vitro study of IVD results. (a) Axial displacement and (b) IDP under compressive load up to 1200 N.

Further comparisons were also carried out to examine the axial displacement and IDP of IVD at L4-L5 vertebral segment. It was found that similar trends were observed in the previous in vitro studies as shown in Figure 4 [26, 27]. The differences between the present FEA results and previous in vitro study results for axial displacement and IDP of the IVD at 1200 N compression load were 7.1% and 6.9%, respectively. Based on these results, the developed FE model could produce appropriate and reliable results for further FE analysis.

### 3.2. The Effects of Human Weight on the Intradiscal Pressure

**Figure 5:**
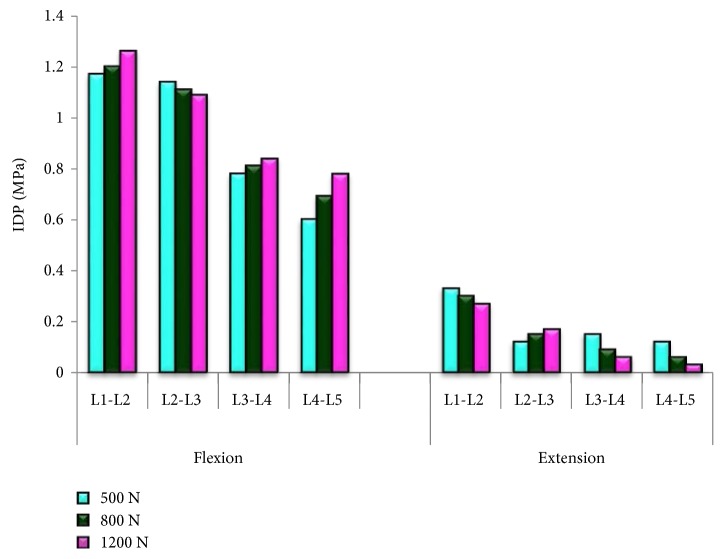
Comparison of the IDP of nucleus pulposus for each IVD vertebral segments in the lumbar spine.

**Figure 6:**
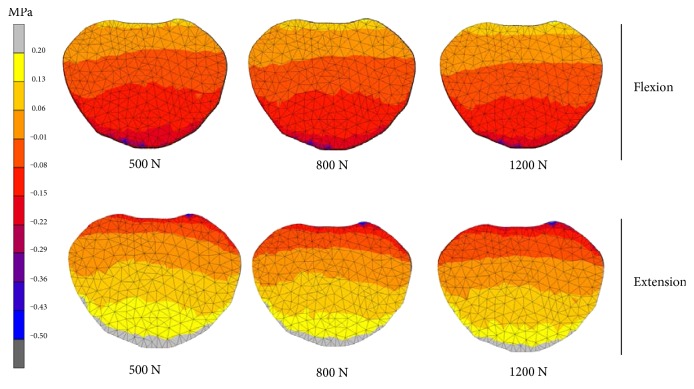
IDP contour plots of nucleus pulposus at L4-L5 vertebral segment.

Figure 5 shows the comparison of the IDP of nucleus pulposus for each IVD vertebral segments in the lumbar spine. It was found that the IDP was increased as the human spine physiological loading increased in flexion motion where the highest pressure was 1.26 MPa at L1-L2 vertebral segment. The IDP was increased in flexion motion but an opposite trend was observed in extension motion. The effects of the human weight were observed to be more significant at the L4-L5 segment as shown in Figure 6. In flexion motion, the 1200 N load generated 30% higher pressure than the 500 N load, respectively, whereas in extension motion, the pressure decreased to 80%. At other vertebral levels, the difference of the IDP between 500 N and 1200 N loads ranges from 4% to 8% in flexion motion, whereas higher range was obtained between 18–60% in extension motion.

### 3.3. The Effects of Human Weight on Annulus Fibrosis

**Figure 7:**
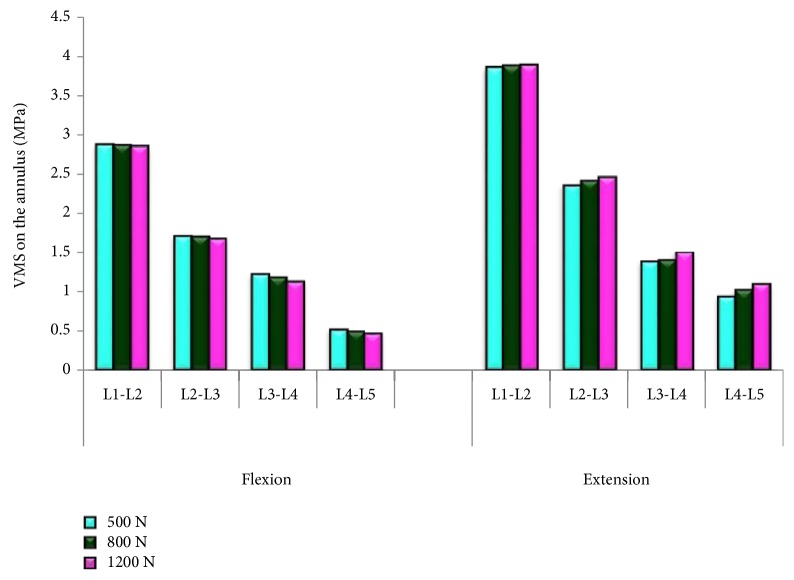
Annulus stress of 500 N, 800 N, and 1200 N loads in flexion and extension motions.

In general, the annulus stress increased as the human weight increased with the maximum annulus stress of 3.9 MPa for 1200 N load at L1-L2 lumbar segment as shown in Figure 7. The highest increment was observed at the L4-L5 where the annulus stress increased to 17% in extension motion, whereas in flexion motion, the annulus stress increased to 10%. At other vertebral levels, the increment of the annulus stress between 500 N and 1200 N loads was between 1.3–8% in extension and 1–8% in flexion motion.

## 4. Discussion

The present study demonstrates that physiological loading of body weight plays an important role of stress distribution at IVD in the lumbar spine. It was observed that increasing body weight will increase the pressure at nucleus pulposus and annulus fibrosis at all levels of the IVD. Furthermore, the position and direction of motion appears to affect these results where the IDP was increased in flexion motion but an opposite trend was observed in extension motion. A severe effect was noticed when heavier individuals continue to experience increased stress and pressure on IVD at all vertebral segments in the lumbar spine particularly at L4-L5 segment in both flexion and extension motions.

The results of the present study show similar pattern to the IDP measured in *in vitro* study where the maximum IDP was found in flexion motion due to the load shift from the posterior towards the anterior of the IVD in flexion motion [12, 28, 29]. The increase of nucleus pressure enhances the tensile stress on the annulus fibers which leads to the excessive stress on the IVD and stimulate the propagation of disc degeneration particularly in nucleus pulposus [21]. This will increase the stiffness of IVD and could reduce its height due to the outflow of fluid through the vertebral body endplates [30]. Subsequently, the fluid loss will increase the proteoglycan and osmotic pressure within the nucleus [30, 31].

The maximum annulus stress was obtained in extension motion due to the load shift from the anterior towards the posterior of the IVD. This phenomenon was also observed in previous clinical study where the structural defect in the vertebral body endplate tends to distribute the load transferred from the nucleus to the posterior annulus. It has been shown that this can potentially lead to pain and could tear the annulus at the disc rim [32, 33].

## 5. Conclusion

Gaining body weight will increase stresses of IVD at all vertebral segments in the lumbar spine particularly the L4-L5 segment. Furthermore, the nucleus pulposus was more severely affected compared with the annulus fibrosus. Although flexion and extension motions of the lumbar spine appear to have different percentage effect on the IVD, it was found that heavier individuals will continue to experience an increase in stress at IVD regardless of the position of the spine. This could be a factor that can lead to early intervertebral disc damage.

## Figures and Tables

**Table 1:** Geometrical parameters of the lumbar spine ligaments [15, 18].

Ligaments	Cross-sectional area (mm^2^)
Posterior longitudinal ligament (PLL)	20.0
Anterior longitudinal ligament (ALL)	63.7
Ligamentum flavum (LF)	40.0
Capsular ligament (CL)	30.0
Intertransverse ligament (ITL)	1.8
Interspinous ligament (ISL)	40.0
Supraspinous ligament (SSL)	30.0

**Table 2:** Material properties of the components in the osseoligamentous lumbar spine model.

Element set	Element type	Material properties	Reference
Cortical bone	3D tetrahedron	*E* = 12,000 MPa, *ν* = 0.3	[16]
Cancellous bone	3D tetrahedron	*E* = 100 MPa, *ν* = 0.2	[16]
Articular cartilage	3D Herman formulation, lower order tetrahedron	*E* = 35 MPa, *ν* = 0.4	[15]
Nucleus pulposus	3D Herman formulation, lower order tetrahedron	Mooney-Rivlin: *C*_1_ = 0.12, *C*_2_ = 0.03	[12]
Annulus fibrosis	3D Herman formulation, lower order tetrahedron	Mooney-Rivlin: *C*_1_ = 0.18, *C*_2_ = 0.045	[12, 16]
PLL	3D truss	*E* = 10.0 MPa (*ɛ* < 11%), *E* = 20 MPa (*ɛ* > 11%)	[15, 18]
ALL	3D truss	*E* = 7.8 MPa (*ɛ* < 12%), *E* = 20 MPa (*ɛ* > 12%)	[15, 18]
LF	3D truss	*E* = 15.0 MPa (*ɛ* < 6.2%), *E* = 19.5 MPa (*ɛ* > 6.2%)	[15, 18]
CL	3D truss	*E* = 7.5 MPa (*ɛ* < 25%), *E* = 32.9 MPa (*ɛ* > 25%)	[15, 18]
ITL	3D truss	*E* = 10.0 MPa (*ɛ* < 18%), *E* = 58.7 MPa (*ɛ* > 18%)	[15, 18]
ISL	3D truss	*E* = 10.0 MPa (*ɛ* < 14%), *E* = 11.6 MPa (*ɛ* > 14%)	[15, 18]
SSL	3D truss	*E* = 8.0 MPa (*ɛ* < 20%), *E* = 15.0 MPa (*ɛ* > 20%)	[15, 18]

*E*: Young's modulus; *ν*: Poisson's ratio; *ɛ*: strain; *C*_1_ and *C*_2_: material constant characterising the deviatoric deformation of material.

**Table 3:** Magnitude of moment loading applied on the lumbar spine.

Loading direction	Flexion moment point		Extension moment point	
	*F_y_* (N)	*F_z_* (N)	*F_y_* (N)	*F_z_* (N)
Flexion	−98 N	−230 N	98 N	230 N
Extension	98 N	230 N	−98 N	−230 N
